# Athletes’ optimal brain care at risk: Should vestibular-ocular motor screening replace established neurocognitive protocols?

**DOI:** 10.17159/2078-516X/2026/v38i1a25038

**Published:** 2026-03-15

**Authors:** AB Shuttleworth-Edwards, VJ Whitefield-Alexander

**Affiliations:** Department of Psychology, Rhodes University, Makhanda, South Africa

**Keywords:** sports concussion, neurocognitive testing, vestibular-ocular motor test

## Abstract

Recent recommendations in South Africa propose replacing validated neurocognitive baseline and post-injury assessment with NeuroFlex, a vestibular-ocular motor test, as part of a proposed new paradigm in concussion management. This commentary examines the clinical, regulatory, and ethical implications of excluding neurocognitive testing and removing neuropsychologist oversight from concussion protocols. Available evidence indicates that NeuroFlex does not currently hold FDA clearance and lacks validation for concussion identification. In contrast, established platforms such as ImPACT and Sway Medical possess FDA clearance and robust peer-reviewed support for concussion assessment. Neuropsychologists play a critical role in interpreting cognitive data, guiding academic and return-to-play decisions, and monitoring longitudinal cognitive health through annual baseline testing, functions not replaced by vestibular-ocular motor measures. Our evidence-based recommendations advocate routine inclusion of post-injury neurocognitive assessment with neuropsychologist application, preferably including baseline evaluation; optional research-based use of Neuroflex in conjunction with cognitive testing; and transparent, scientifically informed consent for all protocol changes.

The management of sport-related concussion in adult and school settings has evolved substantially in the past two decades, driven by recognition of the long-term cognitive and behavioural risks of poorly managed brain injury. Following international trends, many South African rugby-playing schools and sports organisations have adopted automated neurocognitive systems developed for concussion management. Examples include the ImPACT and Sway Medical tests, which are most often administered under the oversight of neuropsychologists. These validated test platforms are employed as a central source of information for medical decision-making and follow-up care. With the neuropsychologist application, the approach can be considered evidence-based and current best practice in multimodal concussion management.^[1–3]^

Recently, however, reports indicate that certain South African sports organisations and independently funded schools have adopted NeuroFlex, a vestibular-ocular motor function test, in place of neurocognitive baseline and post-injury assessment. This shift has been promoted through a webinar proposing a new management paradigm that questions the reliability of baseline cognitive tests and removes neurocognitive testing from protocols.^[4]^ Advocacy for NeuroFlex is based on its alleged ability to assess brainstem function, which is delineated as the probable primary recipient of concussive forces. [*authors’ notes] The webinar further asserts that the adoption of the new paradigm by the Springboks (i.e. the South African national rugby team) establishes this approach as the gold standard for school concussion management. *No published evidence or consensus statement supports such a conclusion, and presenting it as authoritative may risk misleading schools and policymakers*.

In this context, we outline the clinical, regulatory, and ethical considerations surrounding the exclusion of neurocognitive testing and the adoption of NeuroFlex. We also compare NeuroFlex with Sway Medical, which remains widely used internationally, including in South Africa under neuropsychologist oversight.

## Clinical scope and regularity status

Both NeuroFlex and Sway Medical are technologically advanced measurement tools: NeuroFlex uses innovative virtual reality equipment; Sway Medical is a technically advanced 20-minute mobile application.

NeuroFlex was developed in a rehabilitation context to quantify vestibulo-ocular reflexes, generating objective indices of eye–head movement control and associated physiological reflexes relevant to cognitive functioning. While measuring these domains may have value for rehabilitation, the tool currently lacks validation for concussion identification.^[5]^ At the time of writing, NeuroFlex is listed on the manufacturer’s website as *FDA registered*. This is an administrative designation indicating eligibility for commercial distribution, not FDA clearance for concussion management, which requires rigorous regulatory review and application-specific evidence. Accordingly, use of NeuroFlex for concussion should be limited to exploratory research settings until sufficient evidence supports regulatory authorisation for its clinical use in concussion assessment and management.

By contrast, *Sway Medical is FDA 510(k) cleared for concussion assessment in youth and adult sports contexts*, based on rigorous regulatory review and evidence-based validation for that application. It is a multidimensional system encompassing measures of balance, processing speed, reaction time, memory, and executive function with independent peer-reviewed support for valid assessment of core components.^[6,7]^ Since it qualifies as a psychological test, as with the ImPACT test, the Health Professions Council of South Africa regulations (HPCSA Rule 993) restrict its application to registered psychologists, a ruling which accords with the demand for designated professional test use competence by the American Psychological Association (APA, 2010, Ethical Standard 9.07*). [*† *authors notes]*

Notwithstanding these regulatory considerations, the central clinical issue remains: NeuroFlex’s physiological vestibularoptic metrics are not designed to substitute for the differentiated, psychometric measurement of concussion-sensitive cognitive functions required to inform safe return-to-play, learning, or work decisions, when competently conducted by a qualified neuropsychologist. *This limitation is not resolved by pairing NeuroFlex with the SCOAT-6, which includes some unstandardised verbal and working memory tasks but does not provide a validated cognitive assessment framework*.

## Evidence base and scientific validation

A literature search did not identify any independent peer-reviewed research demonstrating the clinical validity of NeuroFlex for concussion use, and no reports on test-retest reliability. Two peer-reviewed studies, published in relevant established journals and involving international authorship from academic, medical, and rehabilitation centres in rugbyplaying countries worldwide, did not support the use of NeuroFlex in this context. Both studies were methodologically robust; although limitations were acknowledged, these were not considered sufficient to undermine the diagnostic findings. Each compared concussed participants with appropriate non-concussed and/or asymptomatic control groups, differing in age range and stage of recovery.

Specifically, Brown et al.^[5]^ conducted a prospective study with 100 elite adult rugby players in the acute post-concussion phase. After querying the construct validity of NeuroFlex and raising the impracticality of administering its numerous measurements on concussed athletes, these authors state that NeuroFlex ‘may not reliably distinguish concussed from non-concussed athletes’ and concluded that ‘current evidence does not support its addition to head injury assessment protocols in elite adult male rugby players’. Treleaven et al. (2023), published in the Journal of Head Trauma Rehabilitation and cited in Brown et al.,^[5]^ conducted a cross-sectional study with 108 youth aged 9–18 years up to 90 days post-concussion. They concluded that although NeuroFlex revealed promise of effective post-concussion eye tracking it ‘is not sensitive enough to detect subtle deficits in children’, posing a risk that ‘children may be prematurely discharged, which puts them at a higher risk of sustaining a second impact or experiencing long-term dysfunction’.

Taken together, these two studies report predominantly non-significant diagnostic findings or statistically significant results in an unexpected direction (i.e. concussed participants performing better than controls). From a conceptual standpoint, such negative or inconsistent findings are unsurprising, given the diffuse, brain-wide pathophysiological processes that characterise the injury. Concussion typically involves fronto-temporal dysfunction arising from acceleration–deceleration and rotational forces, extending well beyond the brainstem or isolated vestibulo-ocular motor pathways. Future research involving NeuroFlex may therefore be more productively directed toward case-based investigation within concussion rehabilitation, targeting individuals with identified oculomotor or vestibular dysfunction. In this context, its virtual reality–based technology may offer clinically meaningful advantages for monitoring recovery within concussion management protocols.

In contrast to NeuroFlex, a robust literature supports the sensitivity, validity, and reliability of computerised neurocognitive screening using ImPACT in concussion evaluation^[8]^, with additional independent, peer-reviewed evidence supporting the validity and reliability of the balance and core cognitive components of Sway Medical for baseline assessment.^[6,7]^ Cognitive testing platforms such as these have been a core component of international concussion consensus statements and position papers for more than two decades.^[1–3]^ An extensive peer-reviewed literature supports their added clinical value and the role of specialist neuropsychological involvement in improving diagnostic accuracy, reducing false-positive findings, and minimising misinterpretation of computerised test data. For example, Van Kampen et al. (2006), published in the American Journal of Sports Medicine and cited in Bauer et al.^[1]^, reported a 29% increase in diagnostic accuracy with the inclusion of neurocognitive testing in a cohort of 122 concussed high school and collegiate athletes assessed two days post-injury. The authors concluded that reliance on self-reported symptoms alone is likely to result in underdiagnosis and an increased risk of premature return to play. Complementing this, Jennings et al.^[3]^ illustrate, through detailed post-concussion case analyses, how specialist neuropsychological involvement mitigates misinterpretation of test data and optimises clinical decision-making.

It is therefore concerning that a proposed “paradigm shift” advocates replacing established concussion management protocols, which include specialist neuropsychological application of neurocognitive assessment tools with demonstrated utility for concussion diagnosis, with a vestibular-ocular motor test that currently has limited validation for this purpose*. Such a shift raises serious concerns regarding the quality of athlete brain care, particularly in contexts where these recommendations have already been implemented*.

## Acknowledging the clinical integrity of cognitive testing

These concerns are further amplified by the rationale offered in a recent webinar-based advocacy calling for the removal of cognitive testing from concussion-management protocols.^[4]^ Central to this argument is the claim that pre-season baseline data lack clinical utility due to developmental change over a one-year interval, particularly in youth populations, and the potential for intentional underperformance. However, this position is inconsistent with the prevailing empirical literature, which supports the clinical utility of baseline and post-concussion cognitive data when interpreted by appropriately trained neuropsychologists. Criticism of cognitive testing has been driven, in part, by untrained individuals conducting simplistic comparisons of baseline and post-injury scores in isolation, overlooking the professional requirement for ecologically and clinically contextualised, individualised interpretation. Moreover, there is no evidence to suggest that baseline data derived from instruments such as ImPACT or Sway Medical are so developmentally unstable as to warrant their wholesale exclusion from neurocognitive evaluation.

Taken together, the literature supports the use of annual baseline testing, beginning as early as 10 to 12 years of age, in conjunction with normative data, with interpretation by neuropsychologists who can integrate both sources while accounting for the inherent measurement limitations of these tools. Standardised cognitive tests employ age-adjusted algorithms and normative datasets that are designed to accommodate developmental change, a process further supported by annual re-baselining. Neuropsychologists evaluate variability within and between standardised broad ability ranges (e.g., below average, average, and above average) and interpret findings in relation to relevant clinical, educational, occupational, and functional benchmarks. They are trained to identify unreliable baseline performance for any reason other than intentional underperformance and to exclude such data from post-injury analyses, drawing instead on alternative interpretive markers. While baseline data represent a valuable adjunct to interpretation, they are not indispensable. Clinically valid norm-referenced post-injury assessment platforms are regularly implemented by neuropsychologists in contexts where baseline testing is not available or is logistically impractical.

Neurocognitive performance is influenced by age, language, and cultural context. In children younger than 10 years, developmental factors may constrain interpretability, while in multilingual or non–English-speaking populations, language proficiency and educational variability can affect test performance, considerations particularly relevant in the South African context. Rather than justifying exclusion, these factors underscore the need for neuropsychological oversight to guide appropriate assessment strategies. Neurocognitive evaluation, with or without baseline data, is a core component of brain injury assessment across age, language, and cultural groups, extending beyond sport-specific applications.

*Accordingly, clinically and ecologically contextualised cognitive assessment, conducted by suitably trained professionals, constitutes an essential and evidence-based component of safe and defensible concussion management, informing individualised return-to-play and return-to-learn/work decisions*.^[1–3]^ Abandoning baseline and/or post-injury neurocognitive testing on empirically undifferentiated or scientifically unsupported grounds risks compromising clinical care and exposing organisations to avoidable ethical and legal vulnerability.

## Transparency and consent for loss of neuropsychological expertise

The shift from an established neurocognitive platform with neuropsychological oversight to NeuroFlex carries significant implications. It removes key objective data that assist sports physicians with return-to-play decisions and eliminates access to specialist scholastic or occupational recommendations. Neuropsychological expertise is essential for interpreting cognitive test results, identifying post-concussion cognitive dysfunction, and guiding academic accommodations or aegrotat applications. Annual baselines using validated digital platforms also enable detection of cumulative or progressive changes following repeated concussions, information critical for advising about continued participation in contact sports. Any removal of these safeguards requires fully informed consent from parents and athletes to maintain transparency and ethical standards.

## Policy recommendations for concussion management

To ensure evidence-based, ethically and legally defensible practice for optimal protection of athletes’ brain health, the following measures are recommended:

1. **Neurocognitive testing with neuropsychologist oversight:** Implement standardised pre-season and post-concussion neurocognitive testing programmes if logistically possible (recommended) or employ postconcussion testing without baseline testing as a clinically valid alternative, for all participants in contact sports, under the supervision of a neuropsychologist.
2. **NeuroFlex for supplementary research application:** Use NeuroFle*x* for an optional research application only until independent, peer-reviewed studies confirm its validity for supplementary diagnostic and/or rehabilitation use in concussion management.
3. **Informed consent for new or revised protocols:** Obtain transparent, scientifically informed consent through communication with schools, sports organisations, athletes and parents whenever concussion protocols are introduced or revised.

## Conclusion

Many South African schools and sports organisations comply with evidence-based concussion management protocols by using validated neurocognitive screening platforms under specialist neuropsychologist oversight. Recently, a webinar presented to schools and sports organisations^[4]^ encouraged moving away from this established framework in favour of a tool of vestibular-ocular motor measurement whose current validation does not extend to concussion management. ^[5]^ Schools and sports organisations are advised to exercise caution when adopting any change in a practice model that differs from professionally endorsed international standards, thereby raising concerns regarding clinical and regulatory defensibility. The adoption of a procedure by an elite professional team does not confer scientific validity, generalisability, or indemnity. This is especially true when used as a replacement for neurocognitive tests robustly validated for concussion assessment,^[6,7,8]^ and when it entails the loss of neuropsychological test application, widely recognised as integral to multimodal concussion care.^[1–3]^ The inclusion of these established best-practice elements is a critical safeguard for athlete welfare and organisational accountability.

## Footnotes

† The term *psychologist* is used in this article in its generic sense, referring to registered psychologists across clinical, counselling, educational and neuropsychology categories, who have two years of formal training and competence in the administration, scoring, and interpretation of standardised psychological and cognitive assessment instruments within their defined scope of practice. This usage is distinct from the term *neuropsychologist*, which denotes a psychologist with additional, specialised postgraduate training and supervised experience in neuropsychology, focusing on the assessment and interpretation of brain–behaviour relationships, typically in the context of neurological, medical, or neurodevelopmental conditions.

* The focus of this commentary is to question the recent webinar promotion of the use of *NeuroFlex* as a substitute for neurocognitive assessment in concussion management protocols.^[4]^ The rationale for selecting NeuroFlex over other vestibular/ocular motor assessment tools was not provided, and this is an omission that warrants exploration beyond the scope of the present article. For instance, the Vestibular/Ocular Motor Screening test (VOMS) is FDA-cleared for use in concussion management when integrated into approved neurocognitive assessment platforms such as ImPACT and Sway Medical. There is substantial evidence in support of this 5–10- minute user-friendly screening, as a reliable, concussion-sensitive, and clinically relevant component of concussion management.^[9]^ As indicated in the article, the innovative virtual reality-based metric of NeuroFlex may hold greater promise for rehabilitation than diagnostic purposes within youth and adult concussion management protocols, once vestibular/ocular concussion effects have been identified.
